# Photoelectrochemical Properties of Graphene and Its Derivatives

**DOI:** 10.3390/nano3030325

**Published:** 2013-07-03

**Authors:** Alberto Adán-Más, Di Wei

**Affiliations:** 1 Department of Materials Science and Metallurgy, University of Cambridge, CB2 3QZ, UK; E-Mail: aa744@cam.ac.uk; 2 Nokia Research Centre, Broers Building, 21 JJ Thomson Ave., Cambridge CB3 0FA, UK

**Keywords:** graphene oxide, graphene, reduced graphene oxide, photocatalysis, photoelectrochemistry

## Abstract

Graphene and its derivatives combine a numerous range of supreme properties that can be useful in many applications. The purpose of this review is to analyse the photoelectrochemical properties of pristine graphene, graphene oxide (GO) and reduced graphene oxide (rGO) and their impact on semiconductor catalysts/quantum dots. The mechanism that this group of materials follows to improve their performance will be cleared by explaining how those properties can be exploited in several applications such as photo-catalysts (degradation of pollutants) and photovoltaics (solar cells).

## 1. Introduction

Global warming and the increment of pollutant concentrations are just two of the environmental issues associated with societal development. One possible way to address those and other existing environmental problems could be through the development of highly-efficient photocatalysts; exploiting processes that are based in solar energy. Semiconductor-based photocatalysis relies on the active absorption of a photon from a semiconductor to create an electron-hole pair. This process depends on the band gap of the material [1]. Then, the excited electron has to be separated from the hole created to avoid recombination. This can be used to generate an ultrafast photocurrent response [2]. The electron can also be used to reduce chemicals in the environment by generating radical species such as hydroxyl radicals; which can initiate, for example, degradation reactions [3].

For a semiconductor to be considered a good photocatalyst, the compound must be photoactive, it must have different electron and hole processes so they do not recombine, it must be able to absorb UV and visible radiation effectively, be photo-stable and be biologically and chemically inert, with the exception of the reaction that it has to catalyze. Besides, in order to be mass-produced, it has to be easy to fabricate, cost effective and non-toxic. Some of the most adequate and traditionally studied semiconductors are TiO_2_, ZnO, CdS, ZnS and Fe_2_O_3_ [1,3]. Nevertheless, those materials have some limitations (e.g., TiO_2_ has a limited photoactivity with the radiation provided by solar light [4]) that can be potentially overcome by the use of graphene and its derivatives.

Within the reviewed literature, some authors refer to reduced graphene oxide (rGO) as graphene (G); however, they have different physicochemical properties, which may affect the obtained results. Therefore, they are differentiated throughout the content of this work.

## 2. Graphene Properties

Graphene, a single layer or few layers of graphite with sp^2^ carbon atoms packed in a honeycomb crystal lattice [5], has unique properties that have been researched for the last decade, since it was first isolated in 2004 [6,7]. In order to exploit these properties, potential applications are being developed. An example of that is photo-detectors and plasmonic devices, which are based on its electrical and optical properties [8,9]. The material has different interesting properties such as large surface area (2630 m^2^ g^−1^) [10], gas impermeability, very high thermal conductivity (>3000 W mK^−1^), and extremely high Young’s Modulus (1 TPa), amongst others [11].

Electrical and optical properties are two of the most novel convenient advances for photocatalysis. Since the electronic structure of a single layer of graphene (SLG) overlap between two conical points in the Brillouin zone, the charge carriers can be understood as mass-less electrons or Dirac fermions [5,12]. Graphene monolayers have an electrical conductivity of (4.84–5.30) 103 W mK^−1^ and charge mobility of ≈200,000 cm^2^ V s^−1^. Besides, charge density can be controlled with a gate electrode and it has ballistic transport (negligible electrical resistivity) [5,13]. It is ambipolar (“charge carriers can be alternated between holes and electrons depending upon the nature of the gate voltage”) and, finally, has anomalous quantum hall effect [5,14]. Regarding the optical properties of graphene, it almost has total transparency. A SLG can absorb a 2.3% fraction of light with a very wide spectral width. Besides, its high operating bandwidth allows to process data at high velocities [2,11]. Moreover, the absorption range can be modified in double-layer graphene by tuning the electrical gating. Therefore, by means of an external gate field, the Fermi energy levels of graphene are changed modifying the absorption properties [2,15].

Finally, chemical properties and chemical modification is also of particular interest in photocatalysis since it enables the adjustment of several of graphene’s properties. The objectives behind functionalization are multiple. For example, the problems encountered by the absence of a gap in graphene (and the consequent absence of photoluminescence) can be solved by widening the band-gap through the coupling between graphene and a substrate. Furthermore, the presence of oxides can vary the properties of graphene, influencing the adsorption and desorption of molecules and, therefore, the chemical reactions. This can lead to an improvement of the catalytic properties of graphene by functionalization [12,13]. The reactivity of graphene is not fully understood yet but in order to generate covalent bonds from pristine graphene, it requires the breaking of a sp^2^ bond. In the adjacent regions to that break point, reactivity is enhanced, as well as the geometrically strained regions [14]. However, it is remarkable that rGO and graphene oxide (GO) have oxygen groups that act as reactive regions.

## 3. Graphene Oxide Properties

**Figure 1:**
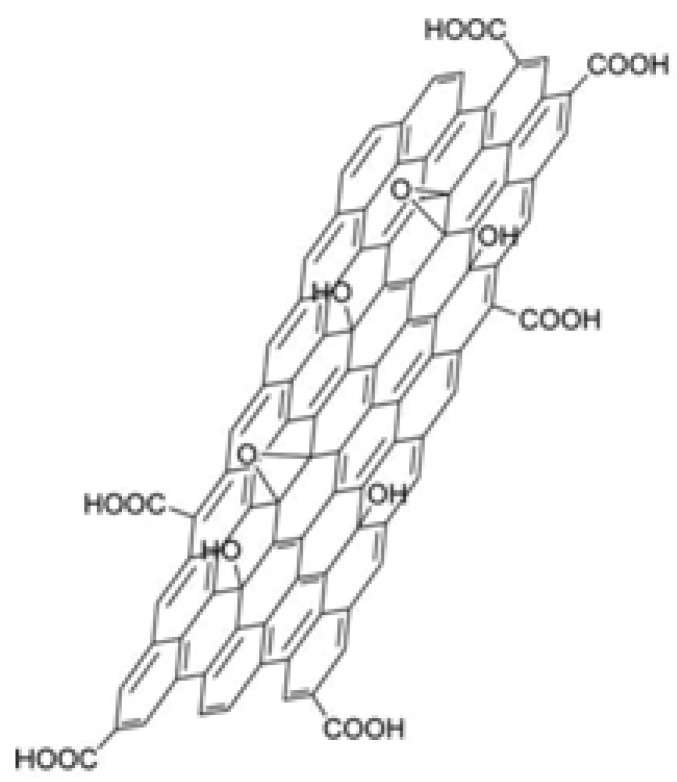
Graphene oxide structure representation. Reprinted with copyright permission from reference [15]. Copyright © 2012, American Chemical Society.

The main derivative of graphene is graphene oxide (GO), which can be directly synthesized from graphite oxide. In this review, we will consider graphene, rGO and graphene oxide-based semiconductor photocatalysers. GO, represented in Figure 1, contains functional oxygen groups (hydroxyl, epoxy, carbonyl and carboxyl) in sp^3^ carbons that vary the properties from pristine graphene [14,15]. Those components are usually the starting point of chemical reactions towards functionalization of graphene. Although the chemistry is still under debate, these oxygen-containing groups provide graphene with hydrophilic character and chemical reactivity [15].

GO also has some other interesting properties. It is an amphiphile with hydrophilic edges that can act as surfactant [16], it is water permeable and ferromagnetic (which is believed to be produced by the defects on graphene structure) [17,18]. Monolayer GO has a lower Young Modulus value than pristine graphene with a value of approximately 207 GPa and a pre-stress oscillating between 39.7 and 76.8 MPa when an average thickness of 0.7 nm of the sample is tested [19]. Moreover, it is an insulating material (the C–O bonds break the conjugation in the lattice, lateral resistivity values of 10^5^ Ω·cm^−1^ [20]) but, by means of a controlled process of deoxidation, an optically and electrically active material can be produced, turning it into a transparent and conductive sample [21]. The vertical resistivity of GO is an order of magnitude lower than the lateral resistivity [20]. These low values of electrical conductivity are maintained in aqueous solutions, where it shows a value of 17 S/m, a very small value compared to reduced graphene oxide in the same conditions (1250 S/m). This proves a restoration of the conjugated system [22].

One interesting property in photocatalyser materials is photoluminescence. In GO, instead of having fluorescence from band-edge transitions (this is the case in semiconductors), the exciton recombination is localized in electronic states with various possible configurations [21]. The advantages of this effect are faster electron transport, lower recombination and higher light scattering; which increase the overall efficiency of the catalyser [20].

Regarding the photoelectrochemical current response achieved with GO, Zhang *et al.* showed that the cathodic photocurrent can be increased by increasing the film thickness, and decreased by UV irradiation. For an average of 9 nm in film thickness, 0.10 µA·cm^2^ of photocurrent density was achieved. It increases to 0.25 µA·cm^2^ for a thickness of 27 nm. Therefore, by controlling the thickness of the film and the time of exposure under UV light, the photoelectrochemical properties of GO can be tuned [23].

A possible explanation is that GO acts as a p-type semiconductor; thus, when it is under illumination the holes tend to go into the GO layer while the electrons are driven to the surface, generating the cathodic photocurrent. Those electrons are captured by the water particles that have been adsorbed on the electrode surface and, after the reaction, they produce hydrogen. The effect produced by UV is related to the behaviour of the oxygen groups and their variation in content. It is also remarkable that the optical band gap of GO is around 3.06 eV and the film thickness nearly has no effect on the optical band of GO [23].The maximum value of capacitance in rGO obtained is 205 F/g with a power density of 10 kW/kg in an aqueous electrolyte with an energy density of 28.5 Wh/kg. Usually, high surface materials in the effective surface area depends on the distribution of pores at solid state. However, this is not the case for reduced graphene oxide. It depends on the number of layers—the fewer number of layer, the less agglomeration and, therefore the best capacitance results [24].

Yang *et al.* coated chalcogenide T4 clusters with rGO to avoid the decomposition of the clusters. This coat not only enabled photo-induced charge separation but also improved by 141% the photoconversion rate of the cluster. Since rGO does not have an energy gap, they are supposed to trap temporary the photo-generated electrons with the consequent reduction of surface recombination. In a Nyquist plot the smaller the radius, the better the charge transfer ability. When rGO was applied the radius was smaller, proving that the separation was more effective and the interfacial charge transfer occurred at the interface of the cluster with rGO. The electrons that are generated in the cluster were captured by rGO and then transferred, avoiding the direct recombination. Besides, it prevented the photo-corrosion. Similar results were found with GO; however, since it is much less conductive, the rate of photocurrent achieved was smaller [25].

Bell* et al.* characterized the photoelectrochemical properties of rGO by using a three-compartment cell comparing the results between rGO/TiO_2_/FTO and TiO_2_/FTO composites. The magnitude of the anodic photocurrent generated by illuminating the film was determined by two factors. First, the speeds at which electrons withdraw from TiO_2_ to FTO. Second, the current lost as a result of recombination within the film and at the film/electrode interface, rGO improved the photocurrent of the system in a factor of 1.5–3 times. Moreover, the transient photocurrent decay (that provides qualitative understanding of the charge recombination behaviour) is increased from 3 to 6 s. This effect reveals that the presence of rGO increases dramatically the lifetime of the electron within the film [26].

By studying the conductance and capacitance of the same system, they determined an optimal ratio for TiO_2_:rGO of 0.7:4 mg, which shows that the conductivity can be prejudiced by light-blocking through rGO. It also facilitates the conduction between the nanoparticles film and the substrate which may be useful to construct a photovoltaic cell that exhibits 10 times more photocurrent [26]. This level of increase is not always achieved; however, there is always a significant enhancement of the photocurrent due to the activity of rGO, for example, from approximately 20 µA/cm^2^ to 38 µA/cm^2^ under UV light in a photoelectrochemical cell. If the photocurrent calculated is normalized, the maximum increment in value provided by rGO in the same system was a 6.5% for Yun *et al.* [27].

Unlike graphene that is hydrophobic, both GO and rGO can be stabilised in water to form stable colloids by means of electrostatic stabilization, without the need of foreign stabilizers. Through zeta potential experiments, the stability of these dispersions has been studied. It is pH dependent and lower than −30 mV at pH greater than 6.1. When the zeta potential reaches a value of −30 mV, it is considered that there is enough repulsion to prove the stability of dispersion. The electrical conductivity achieved for water-dispersed rGO goes up to 7200 S·m^−1^. The tensile modulus is 35 GPa, it is flexible and thermally stable. The resistivity is 2.0·× 10^7^ Ω·m at RT with a transmittance of 96% [28].

It is remarkable that, at the moment, only rGO and not pure graphene flakes can be found in aqueous solution. Therefore, the electrochemical properties are always related to this derivative. The electrochemical potential for reduced graphene oxide is 2.5V in 0.1 M PBS (pH 7.0) while the charge transfer resistance determined from AC impedance is much lower than in graphite and glassy carbon electrodes. The electron transfer behavior and the consequent redox peaks are studied in cyclic voltammetry, which show very well-defined peaks. Besides, the fact that the anodic and cathodic peak currents are linear with the square root of the scan rate indicates that, probably, these redox processes are controlled by diffusion. The ideal peak-to-peak potential is set to be 59 mV. In the case of rGO’s CV, the value is extremely close. This value is related to the electron transfer coefficient, indicating a very high single-electron electrochemical reaction. The value for the electron transfer constant in the edge plane is 0.18 cm/s, much higher than 0.055 cm/s obtained for glassy carbon electrodes in a system with [Ru(NH_3_)_6_]^3+/2+^ as a redox couple. This experience has been tested with other redox couples and it has always indicated that the electronic structure and the surface physicochemistry are extremely enhanced in electron transfer processes on rGO. In the basal plane it is inert electrochemically, with a transfer constant below 10^−9^ cm/s [29]. The electrochemistry of rGO is controlled by its edges. In them, the heterogeneous electron transfer (HET) is fast, which determines the good redox peaks obtained in the electrochemical tests [30].

If rGO is used in cyclic voltammograms as an electrode in 1.0 M LiPF_6_ with Li as counter and reference electrode, the cathodic current generated is similar to graphite with large initial current loss and no anodic current. Nevertheless, in the second cycle it loses all charge capacity retaining only a 12.4% of its original capability. However, the first discharge had a discharge capacity of 528 mA·h·g^−1^ with a cutoff voltage of 2.0 V. The specific energy density was 1163 W·h·kg^−1^. These values show the really promising electrochemical possibilities of this material, which are quite different from graphite [31].

To summarize, graphene and graphene oxide have an extensive surface area, being an excellent substrate for semiconductor particles, excellent mechanical and optical properties. The optoelectronic and chemical properties are the main difference between the two materials. Graphene has excellent conductivity and transparency while GO is a more opaque insulator. However, GO can be either chemically functionalized or reduced to produce rGO. With these materials, a tuneable band gap can be achieved with low recombination rate and high photocurrent response.

## 4. Production

Before dealing with the production of graphene/semiconductor photocatalysts, it is important to understand the different production methods of graphene and its derivatives. They are the key to generating graphene, rGO or GO and therefore, different properties. The first demonstration of isolation was done by Novoselov *et al.* with the “Scotch tape method”, where bulk graphite was placed on the sticky side of regular tape and peeled away. Since that moment, many synthesis procedures to obtain graphene have been developed [7,32,33]. Many groups have already compiled different production methods, such as the work done by Cooper *et al.* and the articles published by Zhu *et al.* and Kuila *et al.* [34,35,36].

One example of graphene synthesis is the photolithographically patterned trenches developed by Frank *et al.* that shear off graphite which is then rubbed on silicon dioxide to produce graphene [37]. Some other examples are molecular beam epitaxial growth on SiC by thermal decomposition [38,39]; solvothermal synthesis (a pyrolysis of an alcohol, usually nano-dispersed ethanol, and an alkali metal (Na) that gives fused monoatomic sheets of graphene) [40]; unzip of multi-walled carbon nanotubes (MWCNTs can be cut longitudinally if they are first suspended in H_2_SO_4_/KMnO_4_); electron beam irradiation of Poly(methyl methacrylate) (PMMA) nanofibres, arch discharge of graphite, thermal fusion of PAHs and conversion of nanodiamond [35].

Large area films of graphene are produced by chemical vapour deposition (CVD) based on the reaction of carbon-based gases on a metal catalyst [32,33]. A metal substrate is placed into a furnace and heated at low vacuum at high temperatures to increase its domain size by annelation. Then, methane and hydrogen gases are inserted into the furnace. Carbon atoms are deposited on the surface of the substrate through chemical adsorption with hydrogen as a catalyst. When the furnace is cooled, it crystallizes into single layer graphene (SLG) [33]. This technique has been developed on top of various metal substrates (Pt, Ni, Fe, Pd and Co). It has also been modified to generate other enhanced synthesis techniques, namely remote plasma-enhanced CVD, surface wave plasma, inductively-coupled-plasma CVD and roll-to-roll production [41,42]. One of the advantages of this technique is the ease to transfer the SLG to other substrates by means of polymer substrates [43].

From the aforementioned techniques, only graphene grown by CVD on different metals and their modifications are, currently, scalable processes. Roll-to-roll technique is a promising technique that can allow sample transfers, produce good quality graphene and can be scaled-up [42,43]. However, since pristine graphene has no functional groups, it makes infeasible dispersion and contact with photocatalysts [10].

Other relevant production methods are based on the obtainment of reduced Graphene Oxide. Thus, GO production methods shall be addressed first. The most important methods of GO synthesis are mainly based on three graphite oxidation procedures. In the first one, KClO_3_ reacts with graphite in fuming HNO_3_. The second method is a modification replacing KClO_3_ with H_2_SO_4_. In the third place, a process generally known as Hummers method, a mixture of KMnO_4_ and H_2_SO_4_ reacts with graphite to form oxide graphite. These three methods of wet chemical synthesis are the basis of GO production [44].

Probably, the most commonly used techniques are variations of Hummer’s method. Raw graphite is oxidized using KMnO_4_:H_2_SO_4_ and NaNO_3_ producing positively charged carbon layers with negative hydrogen-sulphate ions. The two layers increase their distance by hydrolyzing the compounds between the carbon layers. Then, by removing the extra ions produced by the oxidants, the layers tend to separate automatically and thin-film particles in aqueous solution are obtained. After several treatments, uniform-thin graphene oxide films are produced [45,46,47].

Moreover, there are other methods of producing GO; namely, sonication of graphite oxide and RF Plasma functionalization (produces GO by etching the graphite surface and selectively oxidize SLG and the top later of multilayer samples. It is used for photoluminescence and optoelectronic purposes) [35,48,49].

As far as the scalability of GO production techniques is concerned, they have been proved to be efficient in graphene-based semiconductors production. By concrete conditions of the Hummer’s method, (no Na_3_NO_3_, increased amount of KMnO_4_ and H_2_SO_4_:H_3_PO_4_ in a 9:1 mixture) fewer defects, higher yield, equivalent conductivity and no production of toxic gases is achieved. Therefore, this is considered to be the most suitable method to prepare graphene oxide in large quantities [10,50].

Once GO is produced, reduced graphene oxide can be obtained by means of reducing agents. There are two main reaction groups, chemical and non-chemical reductions. The former group is based on liquid-phase exfoliation, an intermediate process between exfoliation and chemical growth where GO obtaining methods are applied with a following chemical reduction [45,46,47]. The reducing agents are varied: hydrazine hydrate [51], NaBH_4 _[52], sodium hydrosulfite [53], iron/HCl [54] and other metals like aluminium , acetic acid/HCl [55] amongst others [34].

A variation of liquid-phase exfoliation is electrochemical exfoliation, a green mass-production technique to obtain exfoliated graphene flakes. By using a mixture of solvents with narrow electrochemical window (e.g., water) and a liquid with large electrochemical window [e.g., room temperature ionic liquid (RTIL)], hydroxyl and oxygen radicals can be produced by the electrolysis of water. Then, the oxygen radicals corrode the graphite anode on defect sites, grain boundaries and edge sites. This induces the separation of the edge sheets and the intercalation of RTIL anions within the sheets. The electrode is expanded and provokes the precipitation, which makes the sheets precipitate, generating a graphene solution. This is a relevant technique since it can produce rGO with reduced sheet resistance (0.015–0.21 KOhm/sq in comparison to 1–100 KOhm/sq obtainable by chemical reduction) and greater transparency (96% *versus* the 80% achievable by means of chemical reduction). This would greatly affect the final photoelectrochemical performance of the material. The reduction and exfoliation level and the size of rGO sheets are controlled by tuning the applied potential and varying the RTIL [56].

Thermal treatment is a low cost method [36]. Some other methods are microwave-induced reduction [57], flash reduction [58] and solvent-assisted thermal reduction [59], but, as in the case of GO, many other production methods are being continuously developed. The objective is to achieve large-scale production methods of quality graphene [34,36].

As far as the interaction between semiconductors and rGO is concerned, the remaining oxygen-containing groups interact with the semiconductor to attach it. The problem is that Hummer’s method produces a large number of defects. Hence, they reduce the recombination probability, so alternative methods are being developed to reduce the quantity of defects produced. Examples of this are solvent-exfoliated graphene and non-oxidative preparation of graphene with a mixture of water and ethylene glycol by an ultrasonic reaction. These methods would be upscalable and are the leading edge towards mass production of quality graphene [10].

## 5. Photoelectrochemical Cells

In order to understand the photoelectrochemical properties of graphene and its derivatives, numerous examples of existing applications are reviewed in this article. However, it is important to clarify that the main subfield considered in this review is photocatalysis, which is only a subgroup within photoelectrochemistry. Thus, we shall also briefly consider photoelectrochemical cells, the other main photoelectrochemical subgroup [60].

A photoelectrochemical cell is a photocurrent-generated device composed of an electrolyte, a photoactive semiconductor electrode and a counter electrode. In the case of irradiation of the interface electrolyte-semiconductor with an energy level greater than the band gap of the semiconductor, electron-hole pairs are generated. The charge in a semiconductor is distributed creating a space charge region that enables the separation of the electron-hole pairs. The minor carriers arrive at the electrolyte while the major carriers travel to the counter electrode by means of a wire to react with the redox couple. There is one main alternative to the traditionally used Si-based solar cells that has been notably improved by any of graphene’s derivatives. This type of photoelectrochemical cells is dye-sensitized solar cells; although there have been also important improvements in quantum dot solar cells which are briefly discussed in another section of this review [60,61].

Dye-sensitized solar cells (DSSCs) will be considered again in this review in solar cell devices. However, the contribution of graphene and derivatives has been remarkable in improving these systems. Therefore, a brief and more general review shall be undertaken.

DSSCs are formed by three main parts. A semiconductor with a dye that is deposited on top of a transparent conducting oxide (FTO, ITO are the most usual), a redox couple in an organic electrolyte (I^3−^/I^−^) and finally, a counter electrode coated with platinum where the redox couple is restored. In this case, the photo-induced electron-hole pair is tightly bonded together, forming an exciton with higher energy than thermal agitation [62]. There are many challenges to overcome such as the suppression of the charge recombination [60].

Graphene can be a substitute of the transparent electrode. A transparency of 70% in the 1000–3000 nm range with a conductivity of 550 S·cm^−1^ was obtained by using rGO. However, the performance of the device was lower than the analogous system with FTO instead [60]. Another possibility is to use graphene as a junction material between the semiconductor particle and the transparent oxide layer. Li *et al.* reported a composite rGO/TiO_2_ on top of FTO, obtaining better performance than the same device without rGO with a PCE from 5.8% to 8.13% [63].

As far as the electrolyte is concerned, the carbon materials can be simultaneously used as charge transporter in the ionic liquid and as a catalyst for the redox couple reaction. Ahmad *et al.* experienced that—when adding rGO to the PMII electrolyte, the light conversion efficiency increased from 0.16% to 2.1%. Moreover, when a mixture of rGO and SWCNTs was introduced, the efficiency increased by up to 2.5% [64].

Graphene is also a good candidate to replace semiconductor oxides and act as a photoanode. The properties that a photoanode should have are highly active surface area, easy fabrication and capability to perform fast electron transport. Since the most important semiconductor is TiO_2,_ most of the work is related to mixtures of this material with some graphene derivative. The DSSC current can be improved obtaining higher PCE, as shown by Nair *et al.*, where it improved from 6.3% to 7.6%. The top value of PCE achieved for this type of solar cells is, approximately, 12% [60,65].

In the last place, graphene can be used as material for the counter electrode. This part dictates the cathodic activity and affects the performance by controlling the restoration of the redox couple. The values sought are a charge transfer resistance lower than 2–10 Ω·cm^2^. Platinum has been widely used, but it should be replaced since it has high cost and secondary reactions, although it has the best performance so far. One of the best possibilities is carbonaceous materials. Lee and his group reported a 3D nano-foam based on graphene grown by CVD. The values obtained for short-circuit current density and open circuit voltage are 12.1 mA/cm^2^ and 0.7 V, respectively. However, the efficiency achieved was lower than the similar system with Askay *et al.* nearly achieved the same value of the reference device [60].

One advantage of graphene is that it catalyses other redox couples so the low redox potential of the iodine-based couple can be overcome. For other redox mediators, Pt is no longer the best counter electrode; graphene nanoplatelets can have a better performance. On the other hand, the inactive basal plane of graphene limits its interaction with the electrolyte and the reactions rake place in the edges. As a consequence, some groups have tried to modify the carbonaceous material with polymers or by doping it with F. A similar efficiency, as with a platinum counter electrode, has been achieved with a polymer modified graphene. These additions of other components also lead to introduce Pt to increase the catalytic activity of graphene. The result achieved was a 7.66% in comparison to the 8.16% obtained for a Pt sputtered electrode [60].

## 6. Graphene and Graphene Oxide TiO_2_ Photocatalysers

The aforementioned photoelectrochemical properties of graphene, rGO and graphene oxide can be used to develop enhanced photovoltaic systems. The need of renewable sources of energy and the growing interest in both photodetectors and graphene composites have lead to produce novel materials to be incorporated and improve existing applications, such as solar cells, organic pollutant decompositors; H_2_ obtaining or CO_2_ reduction [60].

Therefore, after the production of graphene, semiconductors and quantum dot graphene-based composites have been developed, becoming key materials in the functioning and enhancement of those systems. There is a large number of them, for example, quantum dots such as CdS, CdSe, PbS, ZnS, or semiconductors, Co_3_O_4_, Fe_2_O_3_, PbS, TiO_2_, WO_3_, ZnO, ZnS, *etc.*, on G/rGO/GO [66,67,68,69,70,71,72,73]. They all have different properties and therefore, are convenient for different situations. However, TiO_2_ has been widely studied and proved as one of the most interesting photocatalysers since the work of Honda and Fujishima [74]. Its band gap has sufficient energy to catalyse a large number of chemical reactions. It is stable and has a great performance [75], low price and good performance. Moreover, it is chemically inert and nontoxic [60]. Thus, in this review we will mainly consider the photoelectrochemical properties of graphene and graphene oxide throughout the enhancement achieved through TiO_2_ performance as a photocatalyser in several different systems.

There are four main preparation methods to for G/rGO/GO-TiO_2_ compounds. The first and most important is hydrothermal/solvothermal methods. There are many variants of that method but, in general, precursors (GO or rGO, dispersed by means of sonication in an organic solvent, for example benzyl alcohol, or water and a titanium organometallic compound) are loaded into an autoclave and react at high pressure and temperature during several hours. Depending on the growth conditions, rod-shaped TiO_2_ [76], nitrogen-doped graphene with TiO_2_ [77], nanoparticles [78] and other variants can be produced.

Solution mixing is started with an ultrasonic mixing and then UV-assisted photocatalytic reduction of GO [79]. Layer-by-layer rGO-TiO_2_ can be produced by spin-coating graphene oxide and TiO_2_ and posterior UV radiation to reduce GO and attach TiO_2_ [80]. Another production method is Sol-gel preparation, where a titanium precursor is injected through syringe pumps into an oleylamine solution where GO is dispersed. The mixture is then treated thermally to induce the sol-gel reaction [81]. Finally, in-situ growth the salt is mixed with GO and converted to the oxide while GO is reduced [82].

GO/TiO_2_ can be formed in the last three methods by not proceeding with the reduction of GO to graphene. Besides, also different morphological structures can be produced with GO, such as TiO_2_ nanoparticles wrapped in graphene oxide [83]. In both GO and rGO, TiO_2_ can be self-assembled under several conditions, such as in water/toluene interfaces or anionic sulphate surfactants [84]. Self-assembly techniques are based on hydrophobic/hydrophilic interactions and it is a useful method to control the growth of the semiconductor on reduced graphene oxide and graphene oxide’s surfaces [85]. It is remarkable that all the main production methods are based on rGO and not pristine graphene.

There are two main limitations concerning the use of TiO_2_ as a photocatalyser. It has an electron-hole recombination time of 10^−9^ s, with a chemical reaction response of only 10^−8^–10^−3^ s; and it requires UV radiation since it has a too wide band gap (3.2 eV for anatase TiO_2_ and 3.0 eV for rutile phase). Therefore, G/TiO_2_, GO/TiO_2_ and rGO/TiO_2_ composites should be designed to have visible-light catalytic activity [10,82].

There are three many reasons that make of graphene and derivatives an excellent material to combine with TiO_2_. They provide a way to enhance the separation between the electron and the hole that are produced in a photoexcitation thanks to very high electron mobility. They also enlarge the absorption range, including the visible region, in which the semiconductor operates by narrowing the band gap of the semiconductor to 2.8 eV with Ti-O-C bonds and nano-sized Schottky interfaces and acting as a sensitizer (it directly captures visible light). Finally, they also increase the interaction area and adsorption of pollutants and dyes with the photocatalyser by creating a π-π interaction with the delocalized electrons of graphene-based compounds [10,60].

Regarding the mechanism of electron transfer, the TiO_2_ particles have affinity for epoxy and carboxylate groups, where charge transfer is produced by the reduction of those compounds [79]. Oxide groups are required and, as a consequence, pristine graphene would not be suitable in this case. It has been demonstrated that electrons flow from higher to lower Fermi levels. Since the work function of graphene is higher—4.42 eV compared to the conduction band of at –4.21 eV with a band gap of 3.2 eV—graphene could be used as an electron shuttle so electrons will flow from graphene to TiO_2_ in the contact between those compounds in a process known as percolation mechanism [10,76]. Since graphene is not usually the compound that interacts with TiO_2 _but GO/rGO, this may be, experimentally, the mechanism followed by GO/rGO-TiO_2_ composites.

The reaction mechanism is shown in the following [4,10]:

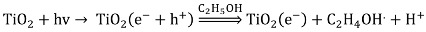
(1)

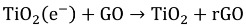
(2)

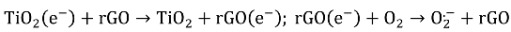
(3)

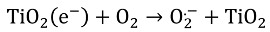
(4)


These electrons are mostly delocalized. Both electrons and holes react with O_2_ and water to form superoxide and hydroxyl radicals, respectively [4]. Thus, the electrons generated can be used to generate photocurrent or produce those radicals that will ultimately react with other compounds, depending on the final aim of the system. In this mechanism, it is shown how semiconductors can take advantage of the good conductivity, adsorption, transparency and chemical properties of reduced graphene oxide.

Not only enhancing the electron-hole pair production is important but also to design ways to produce a more efficient G/rGO/GO-TiO_2_ photocatalyst. In order to do that, Zhang *et al.* studied how to decrease defects and improve interfacial contact between the carbon-based materials and TiO_2_. GO is usually prepared with Hummer’s method, which produces a large number of defects on GO surface. This means that alternative methods to produce this material should be used. The technique used by the group was solvent exfoliation (SEG). SEG/TiO_2_ has, indeed, better properties than GO/TiO_2_ obtained with Hummer’s method by reducing the number of defects but also the number of oxygen groups [86].

Lightcap *et al.* anchored Ag and TiO_2_ to rGO to produce reduction of silver ions into silver nanoparticles. rGO shows excellent properties to store and shuttle electron. Besides, they established that, given the conduction band of Titania at –0.5 V *vs.* NHE and the Fermi level of rGO at 0 V *vs.* NHE, the electron transfer is quick and efficient [87].

Although it seems that rGO/TiO_2_ and GO/TiO_2_ have a promising future in photocatalysis, it still needs more evidence to prove the superior properties of this composite in comparison to, for example, other carbon-based materials combined with TiO_2_ and other semiconductors [86]. However, many efforts for developing enhanced applications with this composite are being made and interesting results have been achieved.

## 7. Applications

The composite TiO_2_ with graphene and its derivatives has large number of applications. We can divide them into three main groups: Quantum dot sensitized and dye sensitized solar cells; degradation of organic, ionic and biologic pollutants and water splitting to produce H_2_ [86].

### 7.1. Water Splitting to Produce H_2_

An efficient way to produce photocatalytic hydrogen from water could be vital in the development of future energy sources. The main problem concerns the fast recombination rate produced in the photocatalyst excitation. Since the semiconductor produces a charge transfer to graphene or graphene oxide, it promotes oxygen and hydrogen splitting. Besides, the increase in the absorption range is again a key contribution of G/rGO/GO in the enhancement of the photocatalytic process. rGO/TiO_2_ was proved to have better hydrogen production than TiO_2_-P25 nanoparticles [88]. Then, a hybrid between them, TiO_2_-P25-rGO, was produced. This material showed a synergetic effect between the different components [71,89].

There are two main reasons attributed to the enhancement of the performance. The first is the close interaction in face-to-face orientation of TiO_2_ and rGO. The second is that the potential rGO/rGO^−^ in comparison to a standard hydrogen electrode (E°(H^+^/H_2_) = 0 V), turns to be −0.08 V; which is less negative than the conduction band of anatase TiO_2_, ≈ −0.24 V. This promotes the flow of electrons from TiO_2_ towards the rGO sheet and reduce a proton producing hydrogen gas [4,90]. However, hydrogen evolution rate is still lower than the state-of-the-art photocatalysts such as lanthanum-doped NaTaO_3_ [82].

Finally, Gao *et al.* conducted an experiment of controlled addition of O_2_ into the atmosphere for controlled production of superoxide radicals. Those molecules can re-oxidize the planar surface of rGO produced by rGO/TiO_2_ and that small quantity of oxygen promotes the hydrogen evolution. Therefore, the utilization of totally reduced graphene oxide is, in this case, unfavourable and the use of partially reduced GO is beneficial [91].

Many composites, besides TiO_2_ with graphene or any of its derivatives have been developed for water splitting, such as Zn_x_Cd_1__−__x_S/rGO, CdS/rGO or WO_3_/rGO. For instance, Jingdong *et al.* designed a WO_3_/rGO photoanode to split water. WO_3_ has stronger absorption and a longer hole diffusion length. The potential of the semiconductor CB is more positive than the H^+^/H_2_ pair. The electrode generates 2.4 times higher quantity of hydrogen and oxygen than WO_3_ on its own and 2.5 higher photocurrent density. The overall photoreaction is limited by the charge separation and it is only effective at a larger bias than 0.7 V vs. Ag/AgCl. In darkness the work function rGO is much lower than that for WO_3_ and, as a consequence, the electrons in the CB cannot be injected. Only through interface states the transfer is possible. However, under illumination, the Fermi energy level in the semiconductor material rises and the electrons can be transferred directly into rGO. The recombination is then avoided and the electrons are quickly transported through an external circuit [92].

### 7.2. Electro-Catalysis: Degradation of Pollutants

**Figure 2:**
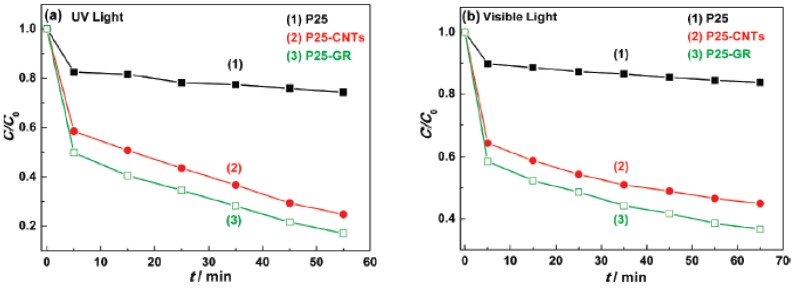
Photodegradation of malachite green (MB) under (**a**) UV light; and (**b**) Visible light (λ > 400 nm) over (1) P25; (2) P25-CNTs; and (3) P25-GR photocatalysis respectively. Reprinted with copyright permission from reference [95]. Copyright © 2010, American Chemical Society.

One of the most important uses of graphene, rGO and GO/TiO_2_ composites is the photodegradation of ionic, organic and biologic pollutants. Kemp *et al.* reviewed the applications of graphene composites for water remediation, where rGO-based Titania compound turned out to be highly useful [93]. Regarding ionic pollutants, rGO/TiO_2_ was proved to have 3.46 times more efficiency in photocatalysis under visible light than commercial P25-TiO_2_ materials. This is produced by the higher conductivity of rGO and the uniform distribution of nanoparticles achieved by the self-assembly technique used [93,94]. The study of rGO/TiO_2_ composite modified with P25 and produced by hydrothermal reaction was also studied by Zhang *et al.* Figure 2 shows that rGO-based photocatalysis results in greater and more selective absorption of the dye, in this case methylene blue. The photocatalytic degradation of malachite green (MB) increases from 20% to 85% with rGO, under a 60-min exposure to UV light. This is 20% greater efficiency than an equivalent composite with CNTs. It also proportions extended light absorption range, due to the narrowing of the band gap and increased efficiency in charge separation and transportation; all three mentioned enhancements induced by reduced graphene oxide [95]. Besides, the reduction of Cr(VI) by up to 91% can be achieved with UV irradiation of rGO/TiO_2_ [93]. Thus, the anchoring of Titania nanoparticles on rGO is a potential candidate for water waste treatment [96].

As far as the eradication of biological pollutants is concerned, *E. coli* was eliminated with GO/TiO_2_ thanks to the properties exhibited by GO, which is biocompatible and antibacterial [93]. After a two-hour treatment in 85 µg/mL of GO, the activity of *E. coli *decreased to 13%. This is produced by the oxygen groups contained in the GO sheet that react with cell membranes creating oxidative stress [97].

**Figure 3:**
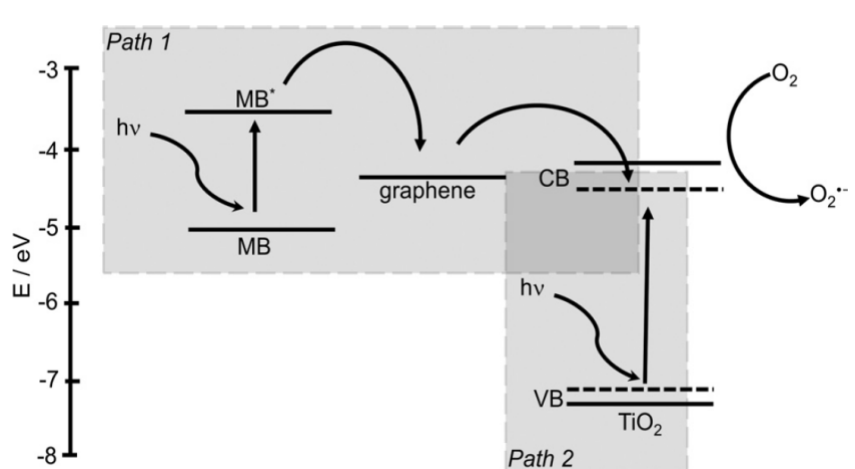
Proposed mechanism for the photodegradation of methylene blue (MB) by graphene-wrapped anatase nanoparticles under visible-light irradiation [10,83]. Reprinted with copyright permission from reference [83]. Copyright © 2012 WILEY-VCH Verlag GmbH & Co. KGaA, Weinheim, Germany.

However, the most important type of compound that can be degraded is organic pollutants. There have been many studies, with different efficiencies, that degrade compounds such as malachite green [98], methyl orange MO [76,77], Rhodamine B (Rh. B) [78], methylene blue (MB) [80,84] and Acid Orange 7 (AO7) [99]. The mechanism behind the degradation, depicted in Figure 3, is similar in all of them. Electrons cannot flow directly from MB to TiO_2_ since their energy levels do not match. A photoexcited electron from MB flows into Titania’s CB via graphene (Path 1), where radical species are generated. Pollutants are usually aromatic compounds that create π-π stacking with rGO, raising the concentration of those molecules near the catalytic semiconductor nanocrystals. The production of oxidants and the reduction of radicals facilitate the reaction when the pollutant is closer. Therefore, the photodegradation is enhanced by π-π interactions. Moreover, as with previous pollutants, large surface area, extended light absorption range, high electron mobility and increased efficiency in charge separation improve the photocatalytic activity [98]. There is an alternative electron mechanism that consists in an electron from the VB of TiO_2_ flowing to the conduction band of the semiconductor. This mechanism is possible by the band gap narrowing produced by graphene sheets. Reactive species that will degrade the pollutant are then produced (Path 2) [10,81].

Some of the pollutants studied are dyes, which are degraded by the use of quantum dot—Titania–G/GO/rGO systems. In these cases, the photoabsorption can be produced by light excitation in the QD or in Titania. That is what Zhang and his work-team reported in a rGO/TiO_2_ composite with PbS QDs. Both PbS and TiO_2_ can get excited by different wavelengths, as a consequence the photocurrent efficiency was increased. The mechanism is analogous to the previous example [100]. Ghosh *et al.* worked with CdSe-rGO-TiO_2_ particles. CdSe, with a band gap of 1.6–1.8 eV can accept visible light. The electrons generated in CdSe are transferred to the conduction band of TiO_2_, whose band gap is 3.0 eV. Besides, rGO can also capture electrons, which are transferred to the CB of the CdSe and, in the same way, to TiO_2_. This is one case in which, by coupling semiconductor—quantum dots and rGO—better photocatalytic results can be achieved [101]. Bi_2_O_3_ is another example of sensitized quantum dot that decorates (001) TiO_2_ facets on rGO. Hou’s group proposed a photocatalytic mechanism. TiO_2_ has a higher reduction potential than H^+^/H_2_ and therefore more active CB edge potential than Bi_2_O_3_. Photoinduced electrons on TiO_2_ are transferred to Bi_2_O_3_ compound and the holes to Titania [102].

An interesting study was conducted by Lin *et al.* and other groups that are researching other types of ternary composites based on rGO-TiO_2_ and Fe_3_O_4_. This photocatalyst can degrade many different organic dyes (RhB, Orange Pure and Acid Blue 92), has enhanced photocatalytic activity, and can be recollected with a magnet. Besides, photodissolution of Fe_3_O_4_ is inhibited, thus, it has a high stability and can be reused many times. However, its catalytic activity is not as good as pure rGO/TiO_2_ and GO/TiO_2_ composites [103].

To conclude, some of the developed systems could be useful in self-cleaning coating. Under UV irradiation for TiO_2_ systems, the photocatalytic oxidation reactions can degrade organic contaminants. In order to be valid for that task, photo-induced catalytic properties play a key role, and rGO/TiO_2_ would be a perfect candidate for that task [104].

It has been shown that TiO_2_ can be very useful in degradation of organic dye pollutants. At the same time, it can also function as an effective charge collection layer for Solar cells.

### 7.3. Solar Cells

Solar cells are one of the applications where the inclusion of TiO_2_ graphene-based composites can enhance the overall performance. At the moment, the most common type of these devices are Si based solar cells; however, alternatives such as dye sensitized solar cells, quantum dot solar cells and organic polymer solar cells are increasing in popularity. The excellent conductivity of graphene, acceptability and mobility of electrons, transparency, wide band tenability and flexibility provided by graphene may improve the state-of-the-art devices [60].

G/TiO_2_, GO/TiO_2_ and rGO/TiO_2_ can be used in both dye-sensitized solar cells (DSSC) and quantum dots solar cells (QDSC). Dye-sensitized solar cells could substitute traditional silicon solar cells in the future, since they have high photon-to-electron efficiency and low cost [105]. Generally, DSSC have a film of dye-sensitized TiO_2_, a conductive transparent electrode a counter electrode and an electrolyte. The dyes are photoexcited and produce an injection of an electron to the semiconductor film. The dye molecules are restored by the electrolyte, which is a redox couple [60].

The conversion rate achieved so far with TiO_2_ electrodes with ruthenium-based dyes is approximately 12%. This low rate is caused by electrons trapping and random pathways. The high specific area and electron mobility of graphene and derivatives may allow longer lifetimes and better conversion rates. Kim *et al.* embedded rGO on the top layers of an inverse opal TiO_2_ structure. C-Ti bonds enhance electron transport, and, therefore, electron injection and collection efficiencies. Besides, the light harvesting efficiency depends on the dye absorption and the optical properties of the electrode film [60,105].

The incorporation of rGO sheets improved the electron lifetime by increasing the chemical capacitance and decreasing the resistance. rGO was used as electron acceptor layer that transports the negative charged particles, which increased the electrical conductivity. The direct contact of the semiconductor structure with rGO induced a reduction in the recombination loss. The fact that the embeddement was limited to some external layers, where the electrons have a higher potential for recombination loss, improved the electron transport. At the moment, a conversion rate of 7.5% is achieved, a 55% improvement over DSSC with pure TiO_2_ [105]. In addition, as it has been said the workfunction of rGO is 4.42–4.5 eV, which is higher than the conduction band of TiO_2_, making easier the electron transport [60,105].

**Figure 4:**
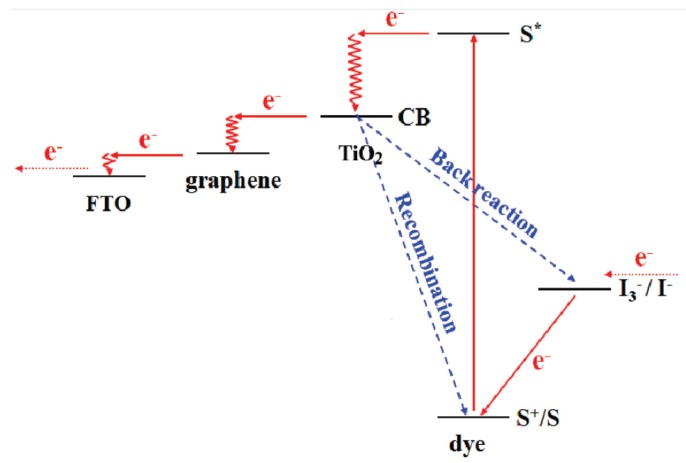
The introduced 2D rGO bridges perform as an electron acceptor and transfer the electrons quickly. Hence, the recombination and back reaction are suppressed. Reprinted with copyright permission from [107]. Copyright © 2010, American Chemical Society.

Figure 4 represents how graphene can act as an electron transport layer, matching the different energy levels. The photoexcited electron produced in the dye is transferred to the CB of TiO_2_. That electron is then transferred to graphene, which acts as a bridge between the semiconductor and the conductive substrate [105].

Tang *et al.* has reported rGO/TiO_2_ on top of ITO as photoanode for DSSC. The increase of electron transport manifested in an increase of the short-circuit current density. They achieved a power conversion five times higher than pure TiO_2_ and conductivity with two orders of magnitude improvement [106].

**Figure 5:**
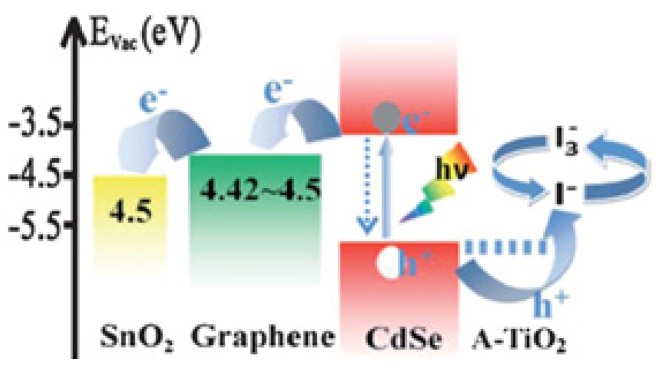
Schematic diagrams of the energy levels in the reduced graphene oxide-CdSe based quantum dot sensitized solar cell [60]. Reprinted with copyright permission from [108]. Copyright © 2011, American Institute of Physics.

Regarding quantum-dot (QD) sensitized solar cells, they have the same structure of DSSC but with inorganic QD (CdS, CdSe, PbS, and ZnS) instead of organic dyes. The advantages of QD in comparison to organic dyes are the high extinction coefficients, tuneable band gaps, large intrinsic dipole moment and good stability. Therefore, the enhancements made with G/rGO/GO-TiO_2_ have the same characteristics of DSSCs’ and the main point would be to improve the electron transport between those QD and the composite. Figure 5 shows this mechanism, similar to DSSCs, used in QDSSCs; where rGO acts as an electron bridge between the quantum dot and the CB of the metal oxide photoanode material [60].

**Figure 6:**
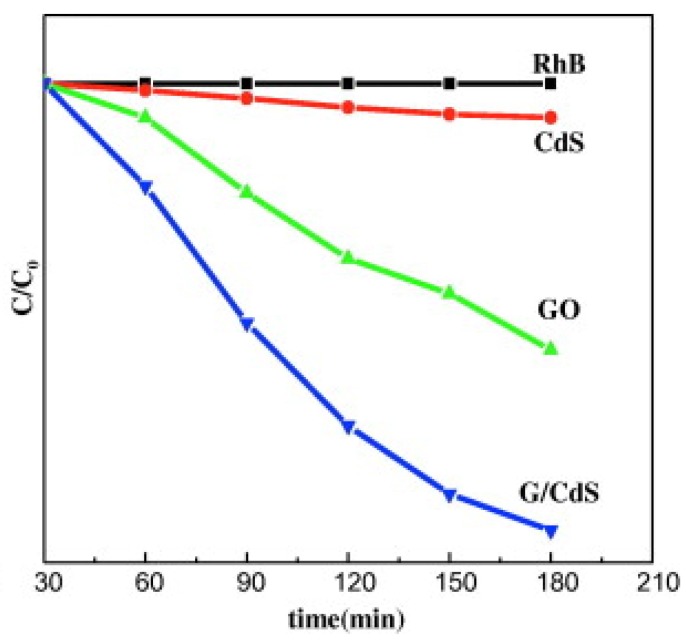
Degradation of Rh. B as a function of catalysis and irradiation time. Reprinted with copyright permission from reference [112]. Copyright © 2013, Elsevier.

**Figure 7:**
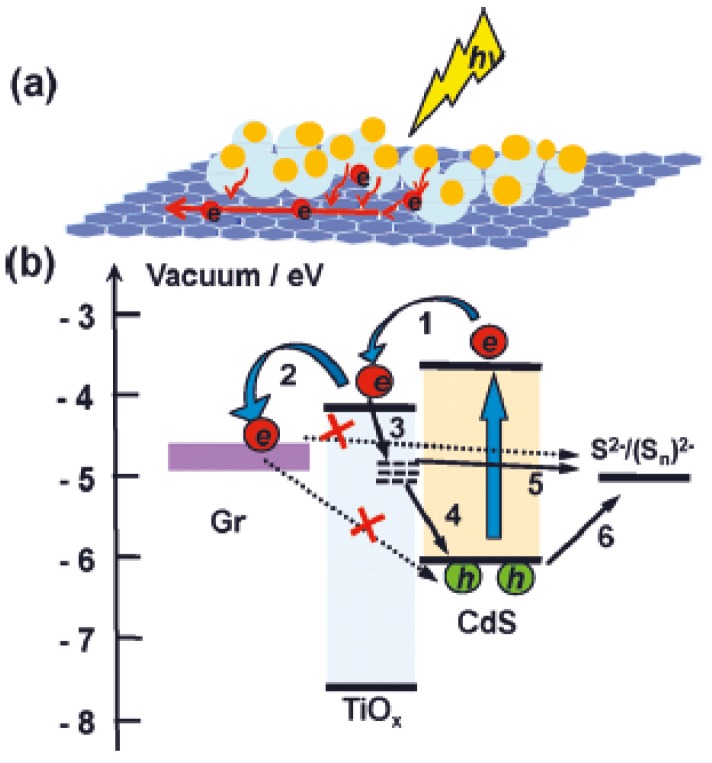
Schematic representation of photo-generated electron transfer processes in a layered reduced graphene oxide/quantum dot (QD) structure with TiO*_x_* interlayer (**a**) and the Energy Band Diagram (**b**) showing the main electronic processes at the interface in QDs: (1) Electron injection; (2) electron transfer; (3) Trapping of the electron at surface states; the two charge recombination pathways of trapped electron recombination with; (4) the hole at the valence band of QDs and; (5) the oxidized redox couple; (6) hole extraction. The recombination between the electron at Fermi level of rGO and the hole at the valence band of QDs and the oxidized redox couple was inhibited by TiO*_x_* layer. Reprinted with copyright permission from reference [111]. Copyright © 2011, American Chemical Society.

For example, a photoanode of TiO_2_ with sensitized Quantum Dots of CdS have been improved by Zhu *et al.* by introducing reduced graphene oxide in its structure. The improvement shown in comparison to the pure photoanode without this material is of 56%. In this case, the conduction band of TiO_2_ (–4.2 eV) was better matched with the work function of the conductive transparent electrode FTO (–4.4 eV) by means of rGO, whose work function is –4.4 eV. This enhanced the overall conduction. Therefore, the caption of an electron by the quantum dot is coupled with Titania, as shown in Figure 6. The semiconductor transports the electron to rGO and this to the electrode. Without rGO, this linear process would not be that direct, since the electrons from Titania could be transported back to the QD in a phenomenon known as back-transport. Recombination of the electrons at the Fermi level of graphene with the holes at the VB of the quantum dot and the redox couple are inhibited by the introduction of an interlayer of TiO_2_ and rGO. However, some electrons are trapped in the surface states and band gap of TiO_2 _and alternative recombination pathways have to be considered (Processes 4 and 5 in Figure 7). Finally, rGO can also absorb energy from visible light, acting as a sensitizer; although the quantity of rGO has to be controlled to avoid light harvesting competition [109,110,111].

### 7.4. Other Applications Based in G/TiO_2_, rGO/TiO_2_ and GO/TiO_2_ Composites

Moreover, there are some other applications of G/TiO_2_, rGO/TiO_2_ and GO/TiO_2_ where the enhancement produced by the carbon-based materials is clear. For example, some developments such as the reduction of CO_2_ [113] and enhancements for lithium-ion batteries (used as an anode in combination with LiFePO_4_ can enhance the cycling performance [114,115,116]) that will define new possibilities for this composite.

## 8. Other Graphene-Based Photocatalytic Composites

It is remarkable that graphene and its derivatives can enhance the photocatalytic properties of different materials, apart from Titania. Good examples of that are quantum dots such as CdS and ZnS and other semiconductors such as ZnO combined with graphene, rGO or GO.

CdS is a photocatalyser under visible light that has a band gap of 2.42 eV, although it self-oxidises liberating Cd^2+^ ions and has a fast electron-hole recombination, which limits its photocatalysis activity. There are several ways to improve the photocatalytic activity of CdS; for example, binding it to other semiconductors or noble metals. Another way is to bind it with a mesoporous or macroreticular material creating a composite were the electrons created by the photoexcitation can move freely while the hole is trapped in the CdS nanoparticles. For that purpose, large surface area and conductive materials are required [1,117]. This could be achieved by using graphene or derivatives, where the efficient electron transport from the semiconductor to the carbon-based material would enhance the photoelectronic response. Nevertheless, studies that combine these two materials are not that common [117].

Zhou *et al.* used solvothermal/hydrothermal method to produce a graphene-based magnetic composite by generating CdS and Fe_3_O_4_ nanoparticles at the same time [118]. In the production of QDs, CdS QDs show photoluminescence responses while rGO/CdS QD do not. This indicates an efficient separation of the electron-hole pair that produces a very strong photovoltaic response [66,68].

Proof of the enhanced catalytic activity is that rGO/CdS exhibits better performance than GO and CdS on their own in the photodegradation of organic and inorganic compounds, such as Rhodamine B, as shown in Figure 6. This fact supports the enhancement of the aforementioned electron transfer, which is used to produce oxygen peroxide radicals O_2_^−^ and hydroxyl radicals OH^−^ by the electron and the hole respectively. Those compounds will ultimately react with Rh. B producing its degradation [112].

It was also used in the degradation of other compounds such as methylene blue, where an efficiency of 94% degradation was achieved by means of visible light; in comparison to pure CdS, that only achieved a degradation of 38% [119]. The amount of rGO or GO is a key issue in the optimal preparation of the photocatalyst [119,120]. Graphene oxide can also be used in degradation activities with CdS. Synthesized by two phase mixing, it can degrade bacteria (*E. coli*, and *B. subtilis*) and Rh. B, methyl orange to produce hydrogen [121] and Cr^6+^ [122].

Another example of graphene’s possibilities would be rGO/ZnS and GO/ZnS composites. ZnS is a II–VI semiconductor with a wide band gap (3.75 eV) that is of interest since it can be used, for example, in field effect transistors, LEDs, photocatalysis and solar cells [123,124]. Recently, quantum dot nanocomposites based on graphene and derivatives have been developed by different production methods such as hydrothermal [123,125,126]; solvothermal synthesis [68,69] or microwave-assisted synthesis [127] amongst others.

The good photocatalytic activity of this compound is a consequence of the rapid photo-excitation, combined with the highly negative reduction potential of the excited electrons [127]. Hu and his group proved the photocatalytic activity of the compound by degrading methylene blue (MB) in water. As expected, rGO raised the electron mobility, acting as an acceptor of negative charge, with good interfacial transfer results that, ultimately, achieved the non-recombination of the pair electron-hole. Besides, another property of rGO that enhanced the photocatalytic activity of the semiconductor is the large surface area, which could disperse the quantum dots so better photon absorption can be achieved and reduces the size of the quantum dots [125,127].

Pan and Liu assigned the charge transfer mechanism to a chemisorption interaction. The photons cause a resonant charge transfer between the semiconductor and the adsorbate, forming specific complexes. Once the electron is excited into the conduction band of ZnS, they interact with reduced graphene oxide and get energy from the excitation levels of this material. In this case, they are then recombined with the holes produced in ZnS leading to better photoluminescence results of the quantum dot. Moreover, if the quantum dots has reduced size, they also have more surface states that can easily coordinate with rGO [125].

Photocatalysis is also possible under visible light. This is a result of a narrowed band gap to 3.45 eV for 5%rGO/ZnS (although it is still too broad to be photoexcited by visible light) and the contribution of reduced graphene oxide. rGO is photoexcited by visible light and the electron is then transferred to the conduction band of the ZnS. Thus, the carbon based material acts as a visible light photocatalyser in a similar way to organic dyes. This behaviour is different to the previously charge transfer mechanism, common for G/rGO/GO-semiconductor composites. Graphene and derivatives can be used, therefore, as photosensitizers [128]. ZnS nanocomposites have been used in many photocatalytic activities, such as photoreduction of CO_2_, water splitting or photoreductive dehalogenation [129]. Graphene, rGO and GO composites with this semiconductor have, therefore, an interest future in photocatalysis, similarly to Titania.

As a final example of the capabilities of graphene, reduced graphene oxide and graphene oxide in photocatalysis, it is interesting to mention their composites with ZnO. This is a semiconductor with a 3.2 eV band gap that is interesting since it is benign to the environment and has a low recombination probability, its valence band is only formed with d orbitals while the conduction band is formed by p-hybridized orbitals [1].

The photocatalytic properties of rGO/ZnO have been proven by many groups through the degradation of methylene blue under UV light [130,131,132]. Zhou *et al.* described the reasons that are attributed to the enhanced performance of the semiconductor in the degradation of the organic pollutant by reduced graphene oxide. As in previous cases, the π-π interaction between the semiconductor and the carbon based material improves the absorbance of MB. The electron ejected from ZnO is accepted by the rGO layer, which was then used to degrade the pollutant. Moreover, the short distance between rGO and zinc peroxide enables the fast transfer of the excited electron [130]. This short chemical bonding reduces rGO/ZnO band gap to 2.90 eV, and therefore, reduced graphene oxide also induces visible-light absorption by the modified electron-hole production process [131].

**Figure 8:**
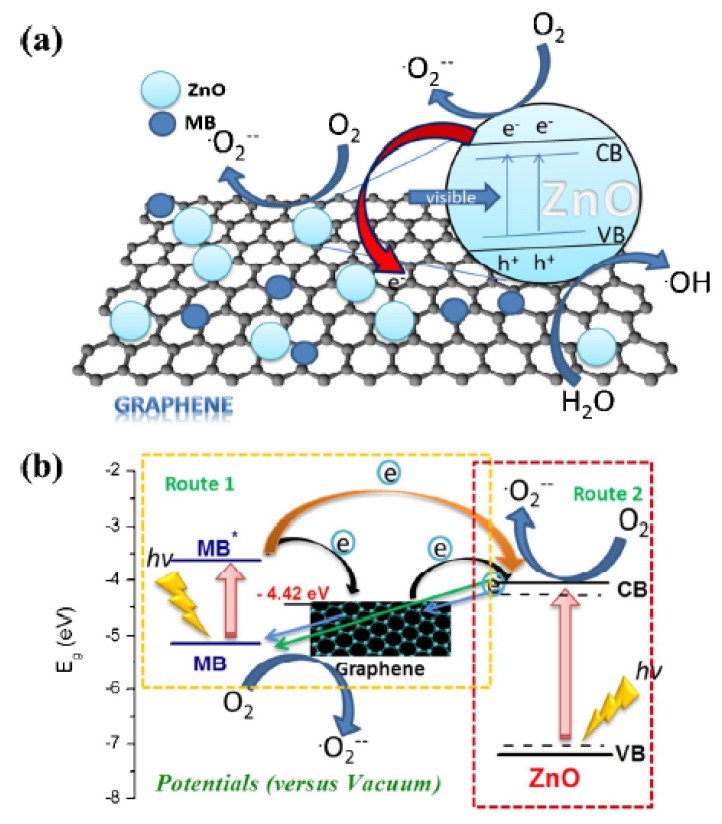
Two proposed mechanisms for the photodegradation of MB by rGO/ZnO composite under visible light and energy diagram of excited MB, graphene and the conduction band of ZnO. (**a**) The excitation of the semiconductor produced by light irradiation generates an electron-hole pair. The reaction with the organic pollutant takes place in the movement of those charges towards the particle surface. (**b**) The dye, which act as a light sensitizer, is excited and transfers electrons. It becomes a cationic radical that self-degrades. Reprinted with copyright permission from reference [131]. Copyright © 2013, Elsevier.

It is interesting the in-depth study of the mechanism of electron transfer that could happen in the degradation of most pollutants done by Ahmad *et al.*, which is depicted in the Figure 8. The dye is excited and acts as sensitizer under visible light which then transfers the electron to the conduction band of ZnO becoming a cationic dye radical, followed by the consequent degradation of the organic compound. The energy level of reduced graphene oxide, −4.42 eV, is lower than the conduction band of ZnO, −4.05 eV. Since MB has a workfunction in excited state of −3.60 eV, the direct transfer from excited MB to ZnO is not possible. However, the reduction in the effective band gap of ZnO by combining it with rGO allows this transference through the reduced graphene oxide layer. Besides, the narrowed band gap allow the absorption of visible light by rGO/ZnO, which also contributes to degrading the pollutant [131]. The enhancement produced by the 2% in weight of graphene in rGO/ZnO produces up to four times more photocatalytic activity than pure ZnO [133].

Since the energy levels of rGO enhance the electron transfer, avoid recombination and allow visible light absorption (although this may reduce the photocatalytic activity by light harvesting competition); several photocatalytic activities have been performed to exploit this effect. For example, as well as other organic pollutants like Rhodamine 6G [134], also metal particles as Cr(VI) have been proved to be reduced with UV light by using rGO/ZnO composite [135]; the fabrication of a fast UV photodetector from rGO/ZnO shell-core structure [136] or the fabrication as electrode materials for supercapacitors with high capacitance values (59 F/g, 61.7 F/g and 146 F/g) and power density (4.8 kW/kg) [137] amongst others. Regarding the activity under solar light, three-component composites have also been developed, like ZnFe_2_O_4_/ZnO immobilized on reduced graphene oxide (ZnFe_2_O_4_ has a narrow band gap of 1.9 eV that allows the solar light caption); which also has magnetic properties that enable an easier separation, and therefore reuse, of the catalyst [138].

### 8.1. Graphene-QD Composites as Photodetectors

Quantum dots combined with a graphene derivative seem to have great potential for future photocatalytic purposes. Within the potential applications, the possibilities provided by their fast photon detection response should not be forgotten. Graphene-based photodetectors have been limited to a photoresponse of ≈10^−2^ A/W since it has weak light absorption and absence of multiple photoexcitation. Nevertheless, its fast response time and broad spectral width are of great interest in photodetection. On the other hand, colloidal quantum dot films have poor carrier mobility and limited absorption range. Graphene, with an absorption range from UV to terahertz range overcomes the “long-wavelength limit”. Besides, the maximum operating bandwidth of photodetectors is restricted by their finite duration of photogenerated current. By creating a composite of monolayer or bilayer graphene with colloidal quantum dots, a responsivity of ≈10^7^ A/W can be achieved by using the high charge mobility on graphene layers [9,139].

**Figure 9:**
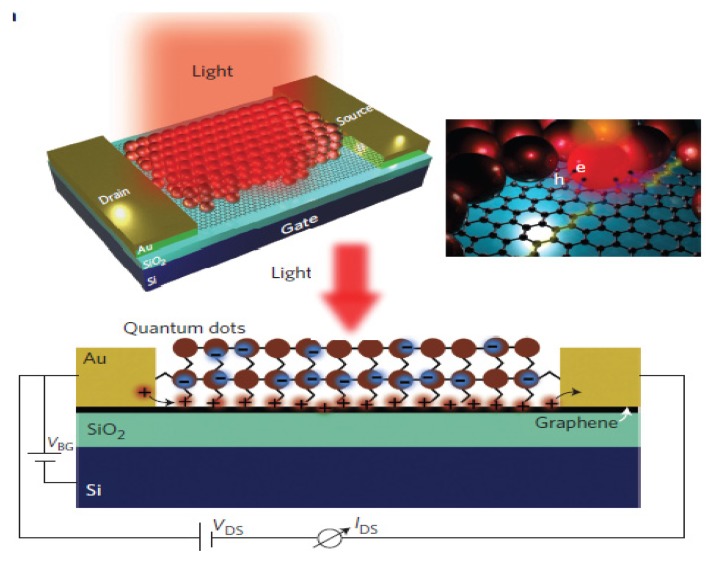
Scheme the hybrid G/PbS phototransistor. Reprinted with copyright permission from reference [139]. Copyright © 2012, Nature Publishing Group.

Konstantatos *et al.* claim that the key of the enhancement in the light absorption of graphene is the implementation of photoconductive gain, i.e., the ability to generate multiple charge carriers with a single photon. Thus, they developed a G/PbS composite with ultrahigh photodetection gain (Figure 9), high quantum efficiency, high sensitivity and gate-tuneable photodetection. The channel of the phototransistor is a monolayer of graphene decorated with PbS QDs that act as a photon absorbing material on top of a Si/SiO_2_ substrate. The functioning mechanism that they proposed is the following: The QDs absorb a photon and creates an electron-hole pair. They are separated at G/QD interface induced by an internal electric field that leads to a band bending, caused by the work function mismatch between the two components [139]. By using an internal field near the quantum dot-graphene interfaces (it can also be done with an external field), effective photocurrent responses can be achieved, with efficiencies up to a 30% of electron-hole separation [9]. The holes are transferred to the graphene layer and the electrons are trapped in the QD [139]. The zero-gap of graphene allows the transmission of the positive carriers through the potential barriers [2]. Positive charges are re-circulated many times, resulting in an overall gain. In summary, the benefits that monolayer graphene provides are gate-tunable sensibility, speed and spectral selectivity [139]. This photodetection gain is relevant in different applications, such as optoelectronic circuits, quantum information technology, biomedical imaging or remote sensing [139].

### 8.2. Other Applications of Graphene-QD Composites

In addition to photodetector systems, the enhancement in optoelectronic properties induced by graphene in these composites is useful in different fields and applications. Zhang *et al.* reviewed the synthesis, assembly, functionalization and applications of graphene-QD composites and highlighted photovoltaic devices, supercapacitors, organic light-emission diodes, fuel cells as a substitute of Pt catalysts for the oxygen reduction reaction and biosensing and bioimaging as other fields where those composites can offer future solutions [140].

## 9. Conclusions

Graphene-quantum dot systems can work as ultrafast photodetector with spectral selectivity. Such an application has a very strict quality requirement on graphene and it is generally using defect-free/less defect monolayer graphene from CVD method. Reduced graphene oxide (rGO) and graphene oxide (GO) have shown great possibilities in composite generation. They can be combined with semiconductors and quantum dots to generate bi and tri-component composites. The wide range of superlative properties of graphene derivatives benefits the photoelectrochemical performance of many materials in multiple aspects. For example, the big surface area with delocalized bonds, in a similar way as “giant” aromatic compound, allows the π-π stacking of several components, which leads to intimate interactions between substrate and organic compounds such as pollutants.

Moreover, the interactions between the oxide groups of GO or rGO and the semiconductor also lead to a close interaction between these two components. This close distance between them enhances the conductivity of photoexcited electrons and reduces the recombination rate of the electron-hole pair. Besides, the high electron mobility of the electron in a layer of graphene also contributes to that effect. This can be useful, for instance, as a bridge between the semiconductor and an organic compound to enhance its degradation.

Many of the most important semiconductor catalysers have a wide band gap that is mainly excited by UV light. rGO narrows the effective band gap of the semiconductor material. It has a wide spectrum of light absorption, thus, it can also act as a sensitizer to capture visible light. However, this mechanism can either lead to enhance the photocatalytic activity or act as a competitor in light harvesting. This would reduce the performance of the semiconductor. A precise control of the parameters and an in-depth study of the reaction mechanisms are required. It seems feasible to introduce rGO and GO in next-generation photocatalytic structures since, a priori; those components lead to enhanced performance results. Although more research is required to fully understand and exploit graphene and derivatives possibilities, current investigation indicates that the performance achieved so far without these materials can be surpassed, and many combinations of different semiconductors, carbon structures, and graphene and its derivatives would be required to take full advantage of this material. By creating new, more complex composites, with tuned coordinated energy band gaps between the different components, we would be able to achieve a high performance photocatalytic response.

However, it is remarkable that, in most cases, it is rGO and not pristine graphene that is used in the development of composites. This is a direct consequence of the different chemical properties of graphene and graphene oxide. The latter allows tailoring and functionalization of the layer, which is much more difficult in monolayer graphene. Nevertheless, it would also be interesting to control the position and concentration of oxygen groups to enhance the optoelectronic properties of graphene, which are more convenient and are affected by the oxygenation of the surface. For that reason, they should be treated as independent compounds and alternative production methods that reduce the number of defects, with intermediate properties, such as electrochemical exfoliation, should be developed.

In conclusion, GO and rGO can be mixed with quantum dots and many other different semiconductors as composites while, on the other hand, pristine monolayer graphene is usually combined with fine-tuned quantum dots to enable a decent photodetector, which uses intrinsic physical properties from defect-free/less defect graphene. Different photoelectrochemical applications will have different requirements on properties and material costs, which is also determined from their different scalability of manufacturing. Graphene and its derivatives obviously provide us various options to explore and it will be exciting to witness how this new type of material will revolutionize/improve the materials used in our daily life.
